# Study on wind load characteristics of stadium under tornado

**DOI:** 10.1038/s41598-023-48999-1

**Published:** 2023-12-07

**Authors:** Zhongya Yuan, Zhibo Zhang, Hao Tang

**Affiliations:** 1 School of Construction and Engineering, Heilongjiang University, Harbin, 150080 Heilongjiang China; 2 School of Civil Engineering, Shijiazhuang Tiedao University, Shijiazhuang, 051132 China; 3 Key Laboratory of Wind Engineering and Bridge Engineering in Hunan Province, Hunan University, Changsha, 410012 Hunan China

**Keywords:** Engineering, Civil engineering

## Abstract

The stadium is a wind-sensitive structure, and the tornado has a greater damage to the stadium. The Ward tornado generating device was used to simulate the wind load characteristics of the stadium during tornado action in order to investigate the wind load characteristics of the stadium. The results of this paper are compared with the experimental results to verify the accuracy of the simulation method. The wind load characteristics of the stadium and its most vulnerable location during a tornado are ascertained by examining the wind pressure coefficient and wind speed distribution law of the stadium’s upper and lower surfaces with different swirl ratio. The influence of uniform wind and wind profile on the wind load of the stadium is explored by comparing and analyzing the wind pressure coefficient of the stadium surface with and without wind profile at the entrance of the wind field. The impact of varying ground roughness on the stadium is compared in order to ascertain how it affects the wind speed, stadium wind pressure, and vortex core radius. The vorticity of the stadium in the tornado wind field with different swirl ratios was compared and analyzed, and the influence of the stadium on the vorticity development of the wind field was explored. This study reveals the effects of swirl ratio, ground roughness and wind profile on the wind pressure, wind speed and vorticity of the stadium under tornadoes, and determines the most vulnerable location and the most unfavorable factors. The above research is helpful to provide reference for disaster prevention and mitigation of stadium structure design.

## Introduction

Tornado is a small-scale vortex generated in strong unstable weather conditions. Its narrow strike range and high destructive force are its key attributes. Compared with other wind-induced disasters, the three-dimensional vortex with strong tornado is more harmful to the structure. More knowledge of the specifics of the tornado's three-dimensional wind field and the way in which the tornado's wind field interacts with the building structure is necessary for structural wind resistance study. In April 2022, a tornado struck the Andover area of central and western Kansas with wind speeds of 73.61 m/s. Numerous structures were totally demolished, and four persons were hurt. In July 2022, tornadoes devastated numerous townships in Jiangsu Province’s Lianyungang area due to strong convective weather. The impact was felt by over 2000 individuals, and the financial losses exceeded 60 million. The lives and productivity of people are harmed by tornadoes. Therefore, it is necessary to explore the mechanism of tornadoes and the impact of tornadoes on building structures, so as to provide a basis for structures to resist tornadoes. Tornadoes usually happen in isolated locations, mainly in rural areas with mostly low-rise structures. People’s demands for spiritual as well as material existence have increased as living conditions have gone up. Rural stadiums are being constructed in farther-flung settlements because they satisfy the population's need for physical activity. Therefore, it is necessary to explore the wind load characteristics of the stadium under the action of tornado wind.

The distance between the tornado vortex core center and the building model and the orientation angle of the building model play an important role in the evolution of the wake vortex and turbulence structure around the building model. The separated flow’s vortex structure and local pressure distribution were altered by the tornado wind field’s curvature. The degree of azimuthal displacement was considered to be determined by the curvature of the horizontal plane flow field around the building. Buildings with low swirl ratio and orientation angles of − 45° and 0° bore the largest horizontal and uplift loads^1,2^. The building’s horizontal and uplift loads were at their maximum, when the building orientation angles reached − 30° and − 45° in a high swirling tornado. The wake flow pattern surrounding the building model and the wind stress operating on it were significantly influenced by the orientation angle of the building model^3^. The primary force generated by the tornado vortex was the lifting force applied to the model of a gable roof building. The wind load operating on the tornado wind was at least three times more than the wind load acting on the gable roof model in the straight air boundary layer wind, for all comparative orientations. The non-Gaussianity of the tornado pressure was thoroughly examined as they examined the external pressure of the TTU building model at various points within the tornado-like vortex^4^. The roof vortex’s skewness and kurtosis were uniformly distributed within a narrow region near the tornado vortex’s center. The vortex’s skewness and kurtosis at various spatial locations differed significantly at the vortex’s core radius.

The ground roughness had an effect on the core vortex of the tornado wind field, so it was necessary to analyze the influence of the ground roughness on the internal pressure of the building model exposed to the stationary vortex. The influence of ground roughness on the local net wind force of the roof was also studied. The internal pressure decreased with the increase of roughness, while the local net roof force increased with the increase of roughness. The internal pressure will fall regardless of the swirl ratio after roughness was introduced since the exterior pressure was less than the internal pressure^5^. Structures with apertures on the windward side wall experience a considerable increase in internal pressure when the ground was rougher^6^. Aerodynamic properties of the tornado-induced wind pressure were clearly identical to those of the boundary layer wind. The boundary layer wind was significantly out of proportion to the suction of the side wall, roof, and leeward wall. The stagnation point and effective wind direction of the windward wall on rocky ground were better than on smooth ground. The addition of roughness led to an increase in the building roof's suction pressure since it enhanced the core updraft^7^. The pressure on the building's surface exhibited a tendency like the low-cyclone tornado flow of the building exposed to smoother terrain, and the difference in pressure coefficients between the building's front and rear edges was lessened. The roughness of the ground increased, and the core radius increased. The ground’s roughness affected the core size reduction at low swirl ratios^8^. The flow field of laboratory simulated tornado was studied by using two-dimensional particle image velocimetry technology^9^. The single-cell or double-cell vortex on the rough surface was observed to change from the multi-cell vortex on the smooth surface with high swirl ratio. There were noticeable variations in turbulence features and velocity due to the increased ground roughness.

The vortex of the two opening types of buildings, the internal pressure of the low-rise building, and the variation in the local net roof wind force were all significantly impacted by the building’s position. The average pressure coefficient in the tornado core diminishes and the standard deviation increased when the opening ratio rose to 8%. The average internal pressure coefficient was the smallest when the building was located at 0.75 times the radius of the tornado and the roof was open on the windward side^10^. A building model with an opening ratio of 0.1% in the vortex core experienced a minimum peak of local net roof wind force that was 1.4 times more than that of a building model with an opening ratio of 3.9%^11^.

The wind pressure distribution caused by the wind load was significantly influenced by the stadium’s design, opening, and wind direction angle. Therefore, it was necessary to explore the wind load characteristics of the stadium in the straight atmospheric boundary layer wind. The impact of wind on the flow field and net pressure coefficient of a large-span retractable roof structure under various roof conditions was investigated^12^. The influence of wind on the retractable roof structure was analyzed, which provided a reference for the wind-resistant design of the large-span retractable roof stadium. The most crucial wind load circumstances were identified, who also assessed the structural reaction^13^. The structural damping response sensitivity with accurate prior evaluation was evaluated. The relationship between urban airflow and indoor natural ventilation was simulated by isothermal CFD simulation method^14^. Neglecting the surrounding environment can result in an up to 96% overestimation of the air exchange per hour, when the simulation results were compared with and without the surrounding buildings. The snow distribution and mutual interference effect of large-span stadium roof were studied by wind tunnel test and numerical simulation. The interference impact between the two structures lessened as building distance increases. The gymnasium's amplification interference effect was most noticeable when the wind direction angle was 45°, which was highly detrimental to the structure’s safety. The most favorable wind direction angle was 270°, and there were both amplification interference and blocking interference^15^.

Currently, there is a dearth of data on stadium damage from tornadoes and a greater amount of research on low-rise buildings in the literature. This study uses a Ward type tornado producing equipment and CFD numerical simulation to confirm the correctness of the tornado field with the wind profile’s entrance. The basic wind pressure, which is mostly influenced by the basic wind speed, serves as the direct design basis for wind load in civil engineering design. The wind pressure on the surface of the large-span stadium is significantly influenced by the vortex motion, which is best described by the vorticity in the wind field. The stadium situated at the center of the tornado was subjected to comparison and analysis using varying swirl ratios for its wind pressure coefficient, ground roughness, wind speed trace, cloud map, and vorticity map. The distribution of wind pressure coefficient on the upper and lower surfaces and the corners of both sides is analyzed, so as to obtain the distribution law of wind pressure on the surface of the stadium under the action of tornado.

## Numerical simulation of tornado

### Turbulence model and governing equations

The turbulence model used in this paper is the shear stress transport turbulence model SST $k - \omega$^16^. This turbulence model incorporates the turbulent shear stress transport process, making it more appropriate for whirling flow and counter-pressure gradient flow when defining turbulent viscosity. The turbulent kinetic energy and dissipation rate equations of this turbulence model are as follows:

1 $$ \begin{gathered} \frac{\partial }{\partial t}(\rho k) + \frac{\partial }{{\partial x_{i} }}(\rho ku_{i} )\; = \;\frac{\partial }{{\partial x_{i} }}(\Gamma_{k} \frac{\partial k}{{\partial x_{i} }}) + \hfill \\ G_{K} - Y_{K} + S_{K} ,\quad \quad \quad \quad \quad \quad \quad \quad \quad \quad \quad \quad \quad \quad \hfill \\ \end{gathered} $$

2 $$ \begin{gathered} \frac{\partial }{\partial t}\left( {\rho \;\omega } \right) + \frac{\partial }{{\partial x_{i} }}\left( {\rho \;\omega u_{i} } \right) = \frac{\partial }{{\partial x_{j} }}\left( {\Gamma_{\omega } \frac{\partial \omega }{{\partial x_{j} }}} \right) + \hfill \\ G_{\omega } - Y_{\omega } + D_{\omega } + S_{\omega } ,\quad \quad \quad \quad \quad \quad \quad \quad \quad \quad \quad \quad \hfill \\ \end{gathered} $$

where $G_{{\text{k}}}$ and $G_{\omega }$ are the mean velocity gradients generated by $K$ and $\omega$, respectively. Valid diffusion terms for $K$ and $\omega$ are $\Gamma_{{\text{k}}}$ and $\Gamma_{\omega }$, respectively.$Y_{K}$ and $Y_{\omega }$ are the diffusion terms of $K$ and $\omega$,respectively. $D_{\omega }$ is the orthogonal divergence term. What is more,$S_{{\text{k}}}$ and $S_{\omega }$ are user-defined terms.

### Tornado generating device

**Figure 1:**
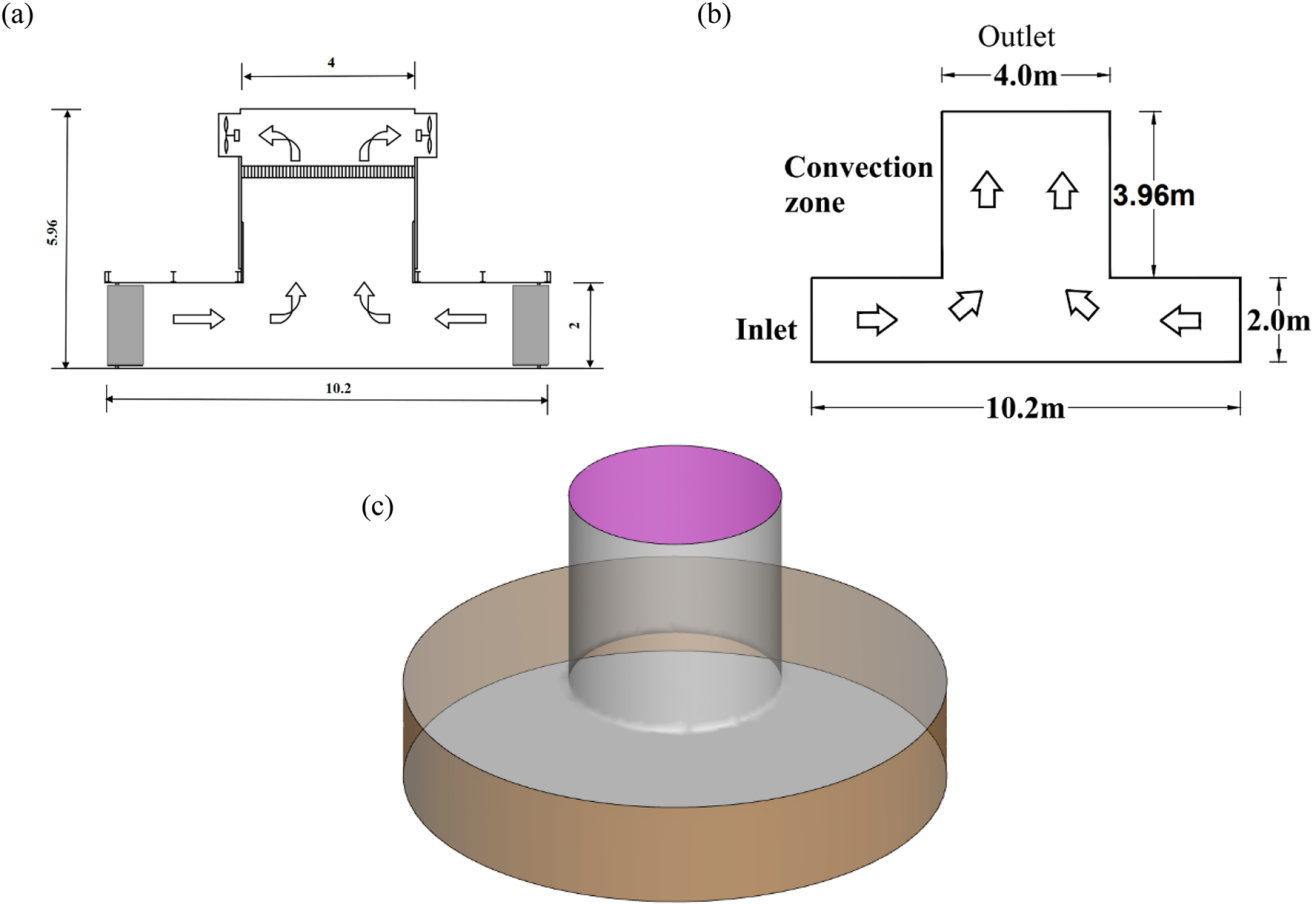
Tornado generating device size, (**a**) VorTECH model size, (**b**) tornado numerical model size, (**c**) generating device schematic diagram.

This paper is based on Ward type^17,18^ tornado generating device to study the wind load characteristics of tornado. We employ the Ward-type tornado generator known as VorTECH^19–21^, that was suggested by Texas Tech University (TTU). The tornado numerical model’s dimensions and schematic diagram are displayed in Fig. 1. The tornado generating device is divided into inflow area, convection area and outflow area. The inflow area has a height of 2.0 m, the outflow region has a height of 3.96 m, and the outflow area has a radius of 2.0 m.

The tangential velocity distribution of the tornado is described by Rankine vortex^29^. The disadvantage is that the curve corresponding to the radius at the maximum tangential velocity is not smooth, but it can better express the general law of the horizontal flow field. The Rankine vortex wind speed calculation expression is as follows:

3 $$ v = \left\{ \begin{gathered} v_{t\max } \frac{r}{{r_{c} }}\quad \quad \;\;0 \le r \le r_{c} \hfill \\ v_{t\max } \left( {\frac{{r_{c} }}{r}} \right)^{\varphi } \quad r_{c} \le r \hfill \\ \end{gathered} \right.\quad \quad \quad \quad \quad \;\quad \quad \;\quad $$

The highest tangential velocity is determined by formula $v_{t\max }$. The radius of the wind speed is $v$. The vortex nucleus's diameter is $r_{c}$. $\varphi$ is the rate of change index of velocity, which is taken as 0.7.

### Wind field simulation and boundary conditions

The dynamics of tornado vortex is affected by vortex ratio and radial Reynolds number. The aspect ratio has an impact on the geometry of the tornado-generating apparatus. The calculation methods of vortex ratio and radial Reynolds number were proposed^22,23^. The calculation formulas of tangential velocity and radial velocity of inlet wind speed are as follows:

4 $$ V_{{{\text{to}}}} = U_{{\text{o}}} ({Z \mathord{\left/ {\vphantom {Z {Z_{{\text{o}}} }}} \right. \kern-0pt} {Z_{{\text{o}}} }})^{{{{1} \mathord{\left/ {\vphantom {{1} {\text{n}}}} \right. \kern-0pt} {\text{n}}}}} {\kern 1pt} , $$

5 $$ S = {{\left( {{{V_{{{\text{to}}}} } \mathord{\left/ {\vphantom {{V_{{{\text{to}}}} } {V_{{{\text{ro}}}} }}} \right. \kern-0pt} {V_{{{\text{ro}}}} }}} \right)} \mathord{\left/ {\vphantom {{\left( {{{V_{{{\text{to}}}} } \mathord{\left/ {\vphantom {{V_{{{\text{to}}}} } {V_{{{\text{ro}}}} }}} \right. \kern-0pt} {V_{{{\text{ro}}}} }}} \right)} {\left( {{{2(H_{{\text{o}}} } \mathord{\left/ {\vphantom {{2(H_{{\text{o}}} } {R_{{\text{o}}} )}}} \right. \kern-0pt} {R_{{\text{o}}} )}}} \right)}}} \right. \kern-0pt} {\left( {{{2(H_{{\text{o}}} } \mathord{\left/ {\vphantom {{2(H_{{\text{o}}} } {R_{{\text{o}}} )}}} \right. \kern-0pt} {R_{{\text{o}}} )}}} \right)}}, $$

6 $$ V_{{{\text{to}}}} = 2V_{{{\text{ro}}}} S({{H_{{\text{o}}} } \mathord{\left/ {\vphantom {{H_{{\text{o}}} } {R_{{\text{o}}} }}} \right. \kern-0pt} {R_{{\text{o}}} }}), $$

7 $$ \tan \theta = {{2SH_{o} } \mathord{\left/ {\vphantom {{2SH_{o} } {R_{o} }}} \right. \kern-0pt} {R_{o} }}, $$

where $V_{{{\text{ro}}}}$ and $V_{{{\text{to}}}}$ are radial velocity and tangential velocity, respectively, and n is 7.$Z_{0}$ and $U_{0}$ are the radial velocity of the reference height and the reference height respectively, and $\theta$ is the size of the inflow angle. $S$ is the swirl ratio^24–26^. $H_{{\text{o}}}$ and $R_{{\text{o}}}$ are the inflow height and outflow radius of the tornado generating device, respectively. The boundary conditions and solution parameters are shown in Table 1.

**Table 1:** Boundary conditions and solution parameters.

Inlet	Velocity inlet
Outlet	Pressure outlet
Ground	Wall
Sidewall	Wall
Model surface	Wall
Calculation	Transient
Turbulence model	SST $k - \omega$
Discrete format	Second-order upwind
Convergence precision	10^–4^
Air density	1.225 kg.m^–3^
*V*_tmax_	Maximum tangential velocity of core radius
*R*_max_	Maximum core radius
Scale ratio	1:100
*S*	Swirl ratio
*K*_s_	Roughness height

## Grid division and simulation method verification

### Tornado wind field simulation and reliability verification

**Figure 2:**
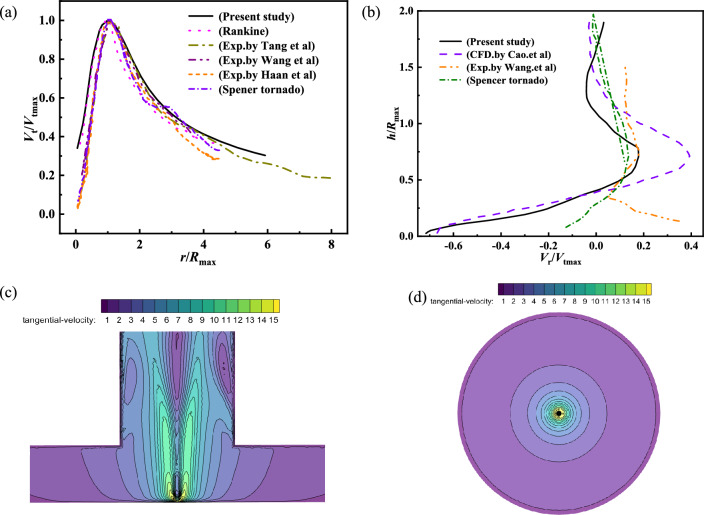
Normalized radial distribution of tangential velocity and radial velocity of tornado wind field, (**a**) normalized radial distribution of tangential velocity, (**b**) normalized radial distribution of radial velocity, (**c**) tangential velocity cloud map of longitudinal section of tornado wind field, (**d**) tangential velocity cloud map of transverse section of tornado wind field.

The simulation results of the swirl ratio *s* = 0.18 and the inlet tangential wind speed 0.43 m/s are compared with the experimental results^23,27,28^ and Rankine model^29^ were fitted with the measured results in order to confirm the accuracy and reliability of the simulation results. The normalized radial distribution of the tornado wind field's tangential and radial velocities is displayed in Fig. 2. The tangential velocity away from the core increases rapidly with the radius direction, and increases to the maximum at the core radius. The tangential velocity gradually reduces along the radius when it is outside the vortex core radius. The simulation results are bigger than the experimental values within the radius of the vortex core. The overall trend from inside to outside is consistent when the simulation findings are compared with the observed and experimental results in this work^27^. The simulation results were compared with the results^30^ is more consistent with the experimental results. The simulation results are in good agreement with the experimental results^27^ when *h*/*R*_max_ = 1.21. The pressure drop and variable tangential velocity components are the main causes of the discrepancy. The tornado wind field is funnel-shaped, with a single vortex core, and its tangential velocity tends to zero, according to the tangential velocity cloud map of the longitudinal section of the tornado wind field. The greatest tangential velocity is displayed as the radius grows until it reaches the core radius. Ultimately, the tangential velocity steadily drops as the radius grows. The tornado wind field's maximum tangential velocity steadily rises due to centrifugal force, as observed by the cloud diagram of tangential velocity at various tornado heights. The simulation results presented in this study are consistent with the observed change trend, the Rankine model, and the experimental verification, thereby confirming the validity of the numerical simulation approach.

### Method validation and grid independence analysis

**Figure 3:**
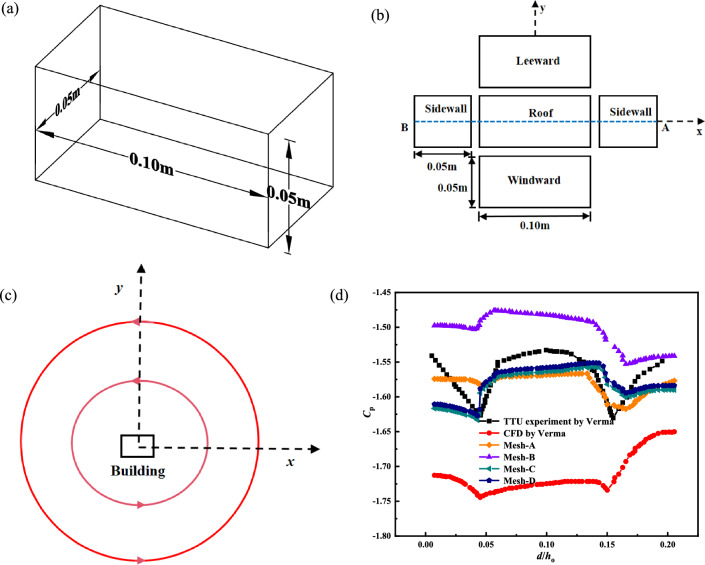
The model is located at the core of the wind field to verify, (**a**) the size of the building model, (**b**) the display of the wind pressure coefficient *C*_p_ profile along the center line of AB, (**c**) the indication of the building model located in the wind field, (**d**) the comparison of the wind pressure coefficient of the model located at the core of the wind field.

The wind pressure on the building surface is greatly influenced by the interaction between the wind field and the building structure during a static tornado. The verification of the model in the core position of the wind field is shown in Fig. 3. The scale ratio of the low-rise building is 1:100. We used a Ward-type tornado generator proposed by Texas Tech University (TTU). The experimental model in the tornado generator and simulator we used was constructed in the same proportion according to the 1:100 model of the wind engineering research field laboratory building of Texas Tech University^26,31,32^. The size of the building model is 0.1 m × 0.05 m × 0.05 m, and the wind pressure coefficient of the building center line AB is taken. The various meshes and their sizes taken into consideration in the mesh convergence study are displayed in Table 2^24,26^. The results of this study are compared with the simulation results^26^ and the experimental results of TTU^33^, when the swirl ratio is 0.83. The simulation results Mesh-C in this paper are close to the experimental results in the overall trend of wind pressure coefficient, and the values are similar. The main difference is that the wind pressure coefficient of the roof is 1.3%. The wind field is impacted by the various shear velocity and roughness components close to the ground. The above results verify the accuracy and reliability of the simulation method again.

**Table2:** Different grids considered for mesh convergence study with their mesh sizes.

Grid	Total elements	Total nodes	First layer grid height	Largest size of grid	Time step sizes
Mesh-A	2,201,117	373,796	0.4 h	4 h	10^–3^
Mesh-B	2,454,153	416,434	0.2 h	4 h	10^–3^
Mesh-C	2,696,815	458,238	0.18 h	4 h	10^–3^
Mesh-D	3,256,354	553,537	0.16 h	4 h	10^–3^

**Figure 4:**
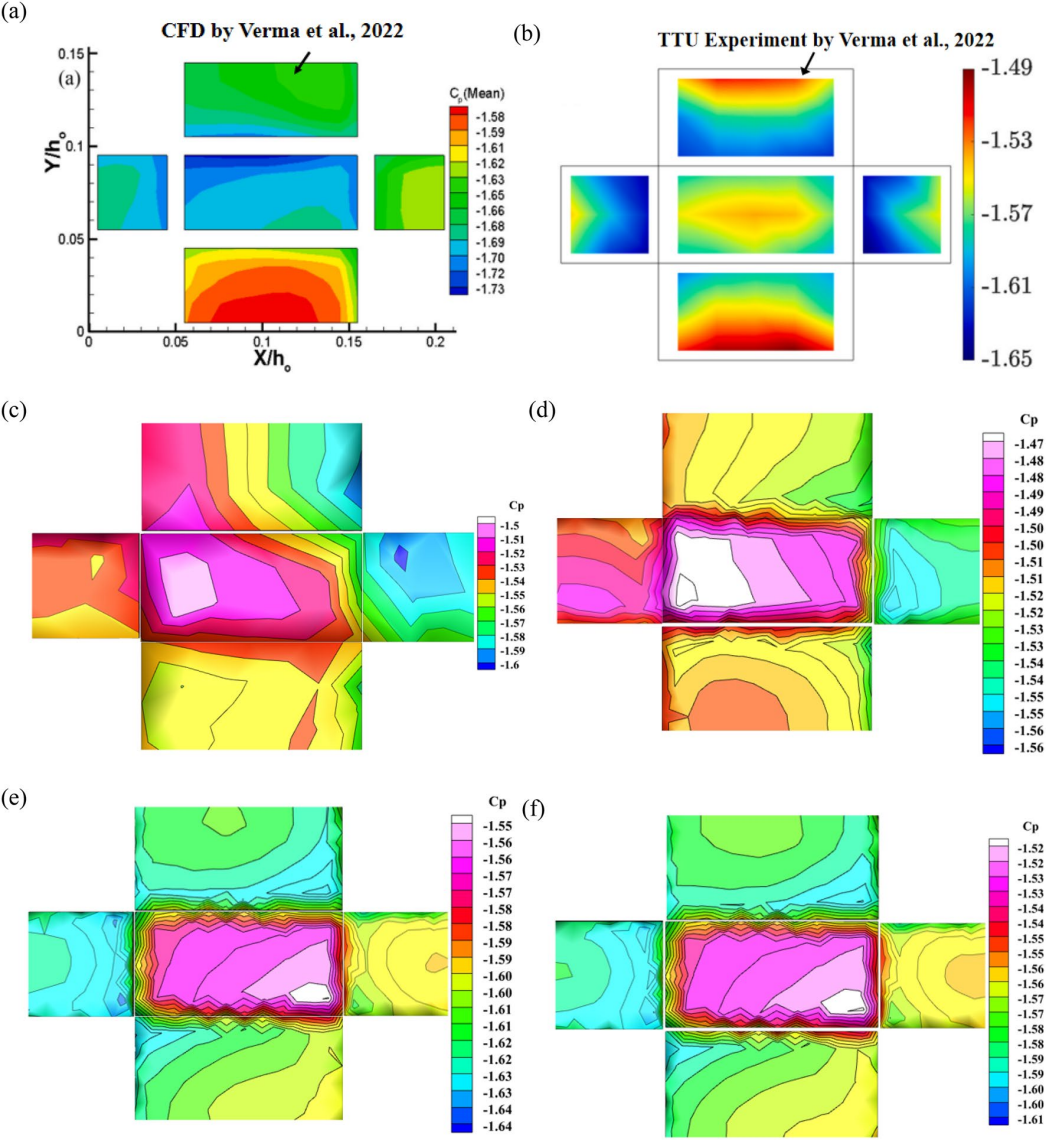
Comparison of the wind pressure coefficient contours of the model at the core of the wind field, (**a**) CFD by Verma simulation, (**b**) TTU experiment by Verma, (**c**) Mesh-A, (**d**) Mesh-B, (**e**) Mesh-C, (**f)** Mesh-D.

Figure 4 shows the comparison of the wind pressure coefficient cloud images of the model at the core of the wind field. The wind pressure range of CFD simulation by Verma is from − 1.58 to − 1.73, and the wind pressure coefficient range of TTU experiment is from − 1.49 to − 1.65. The simulation results of this paper show that the average wind pressure coefficient of Mesh-A is in the range of − -1.5 to − 1.6, the wind pressure coefficient of Mesh-B is in the range of − 1.47 to 1.56, and the wind pressure coefficient of Mesh-C is in the range of − 1.55 to 1.64. Mesh-D simulation results are very close to Mesh-C. It can be seen that Mesh-C has little effect on the simulation results with the change of the number of grids, and the simulation results are no longer affected by the size and number of grids. The results of Mesh-C and Mesh-D in this paper are close to the experimental results, and the deviation is about 0.3%. The variation results from a change in the wind field pressure brought on by a variation in the tangential velocity component. It can be seen that the results of this paper are better.

### Tornado generating device and building model meshing

**Figure 5:**
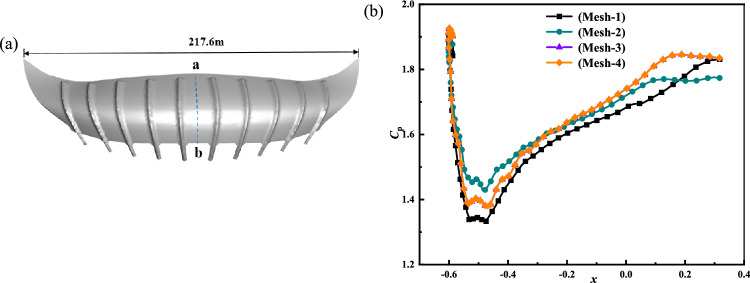
Stadium monitoring position and division of different grid size wind pressure coefficient, (**a**) Along the ab center line of the stadium monitoring point display, (**b**) Different grid division of wind pressure coefficient.

**Figure 6:**
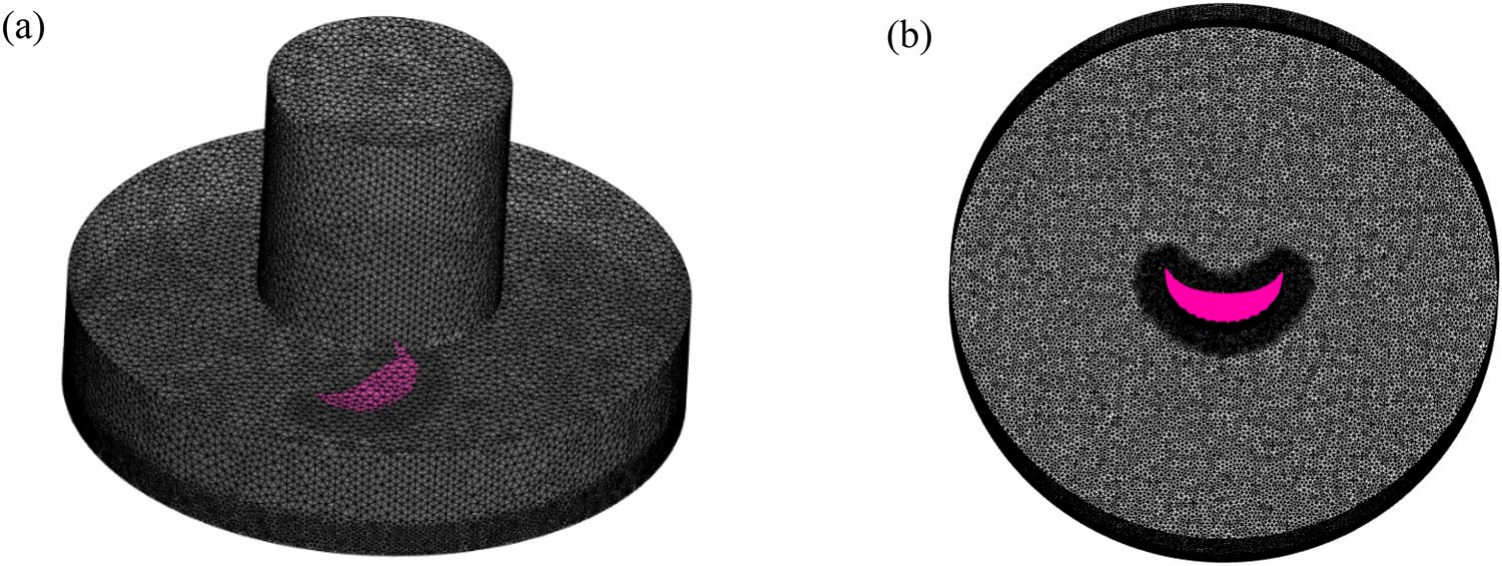
Tornado generation device grid division, (**a**) Tornado generation device grid division diagram, (**b**) Stadium model grid division diagram.

The division parameters of the various stadium canopy grid sizes are provided in Table 3. The stadium’s intricate structure makes it challenging to implement the structural grid division, and the grid’s quality is poor. The usage of unstructured mesh yields high-grade mesh quality. Unstructured grid division can better display the characteristics and local details of complex large-span stadiums. The grid is encrypted close to the stadium and the ground because of the significant changes in air flow caused by these locations. Figure 5 shows the monitoring position of the stadium and the wind pressure coefficients of different grid sizes. The simulation results of Mesh-1 and Mesh-2 are unstable. The simulation findings are identical when the first layer's mesh size is decreased to Mesh-3 and Mesh-4. It can be seen that Mesh-3 is the critical point of meshing. Mesh-3 mesh size was utilized in this study to maximize utilization time and guarantee the accuracy of the simulation results. The grid division diagram and tornado generating apparatus for the stadium model are displayed in Fig. 6. Radical Reynolds number is denoted by *R*_er_. The volume flow of the device in this study is 42.92m^3^/s, and *Q* is the volume flow of the inlet height. *V* is the kinematic viscosity of air, and the value is 1.5 × 10^–5^. The radial Reynolds number *R*_er_ = 4.56 × 10^5^ is obtained by calculation. It is found that when the radial Reynolds number *R*_er_ ≧ 10^5^, the influence of the tornado generating device on the tornado can be ignored^22,34,35^.

8 $$ {\text{R}}_{{e{\text{r}}}} = \frac{Q}{2\pi V}. $$

**Table 3:** Different grid size division parameters of stadium canopy.

Grid	Mesh-1	Mesh-2	Mesh-3	Mesh-4
Total elements	2,478,516	3,683,892	4,263,068	9,105,986
Total nodes	419,114	623,122	750,593	1,536,606
First layer grid height (m)	0.05	0.04	0.038	0.02
Largest size of grid (m)	0.05	0.04	0.038	0.02
Time step sizes	10^–3^	10^–3^	10^–3^	10^–3^

## Numerical simulation of wind load on stadium under tornado

**Figure 7:**
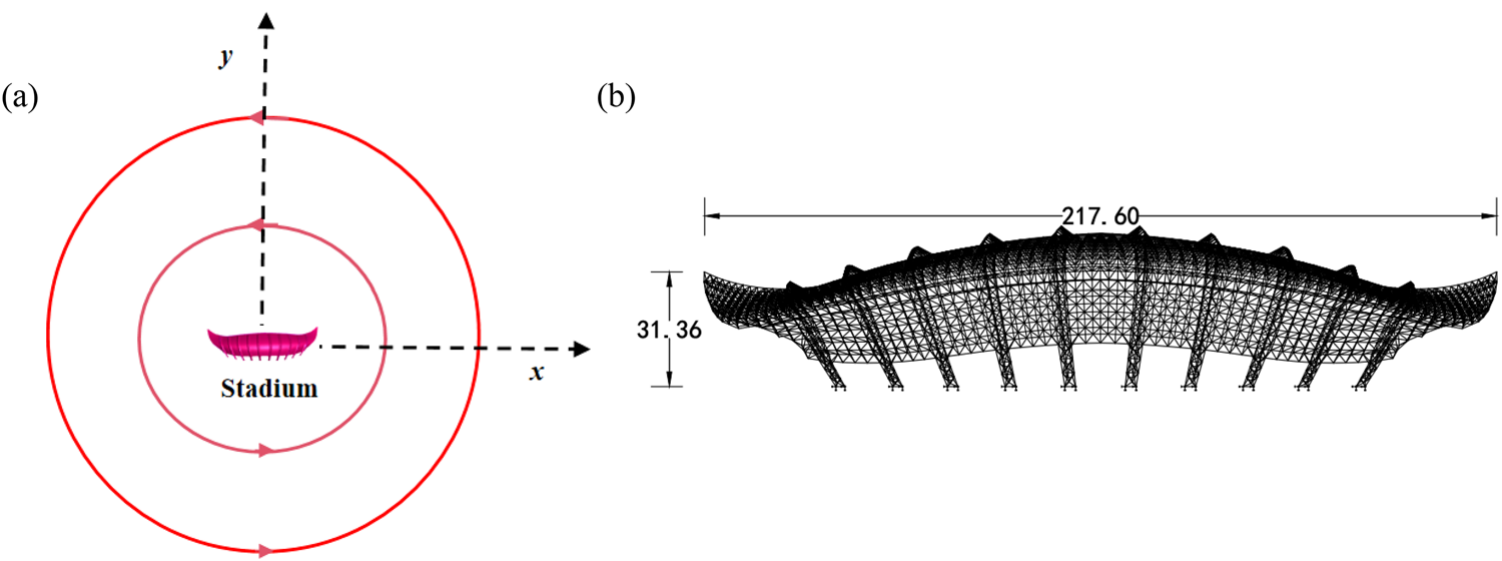
The stadium model is located in the wind field position and size diagram, (**a**) the stadium is located in the wind field position, (**b**) the stadium size.

The complex large-span stadium is simulated and analyzed in this paper. The stadium is 217.6 m and the canopy width is 93.13 m. The stadium's greatest cantilever length is 33.77 m, and its ends are 31.36 m above the floor on either side. The stadium model is placed at the core of the wind field in a ratio of 1:100, and the wind pressure coefficient and vorticity of different vortex ratios and ground surface roughness are explored. The stadium model is located in the wind field position and size as shown in Fig. 7.

The dimensionless wind pressure coefficient^26^ is used to examine the surface wind pressure in order to quantitatively express the stadium’s surface wind pressure. The expression is as follows:

9 $$ C_{{\text{p}}} = \frac{{P - P_{{{\text{ref}}}} }}{{0.5\rho v_{{{\text{tmax}}}}^{2} }}, $$

where $P$ is the measurement point wind pressure, $P_{{{\text{ref}}}}$ is the reference point static pressure, take a standard atmospheric pressure. The air density is $\rho$, and the highest tangential velocity is $v_{{{\text{tmax}}}}$.

### Comparison of wind pressure on stadium surface with different swirl ratio

**Figure 8:**
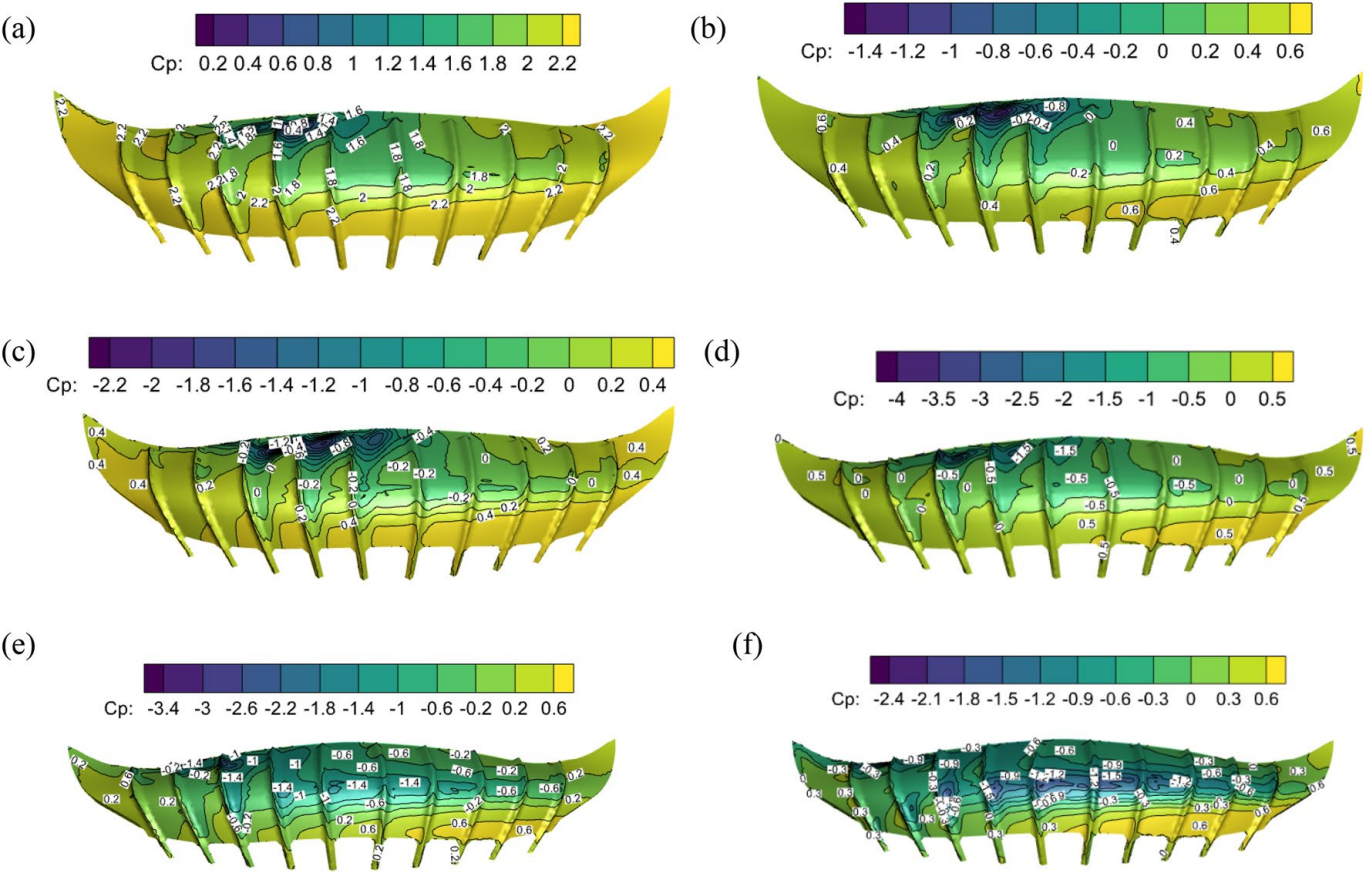
The wind pressure coefficients on the upper surface of the stadium with different swirl ratios, (**a**) *s* = 0.2, (**b**) *s* = 0.4, (**c**) *s* = 0.6, (**d**) *s* = 0.8, (**e**) *s* = 1.0, (**f**) *s* = 1.2.

**Figure 9:**
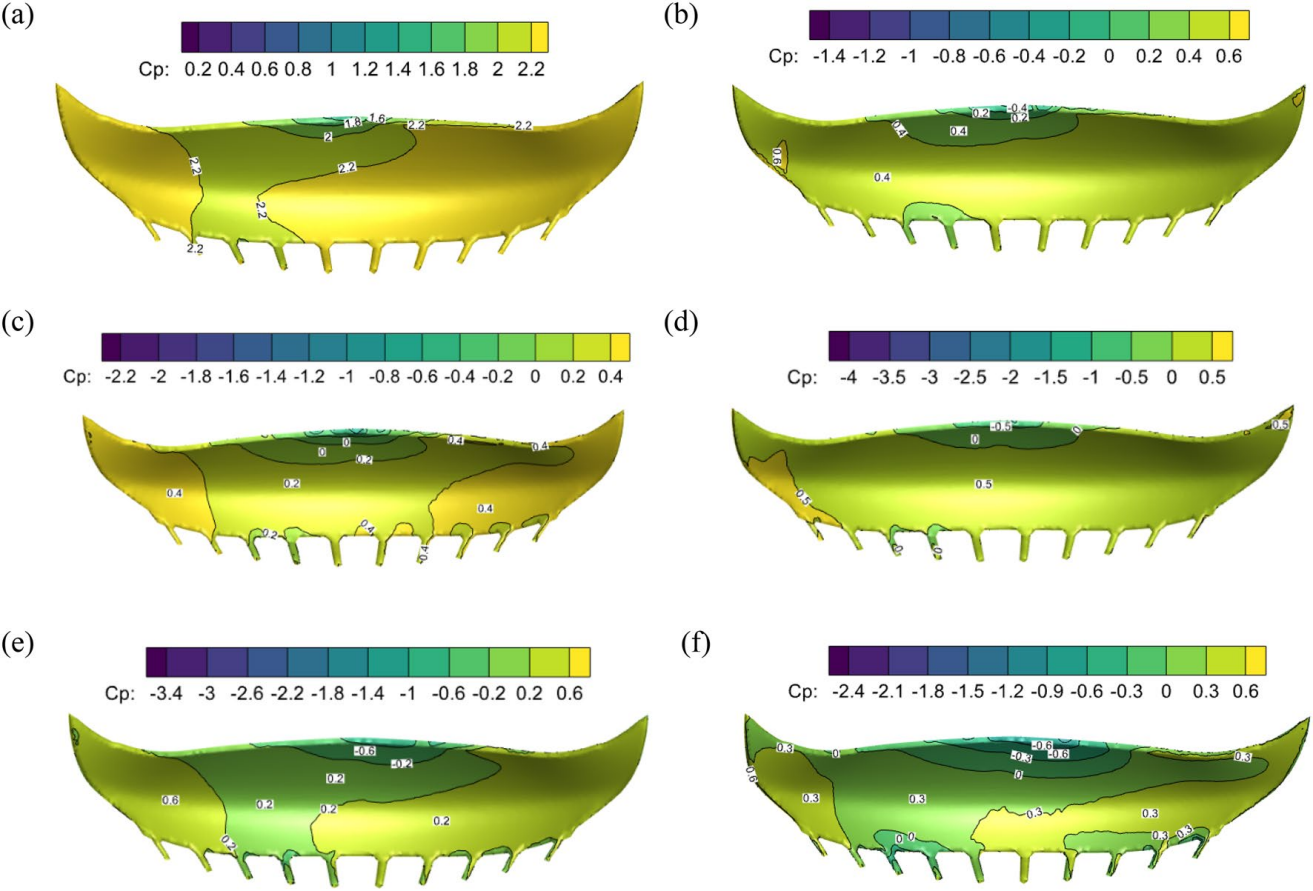
The surface wind pressure coefficients of the stadium with different swirl ratios, (**a**) *s* = 0.2, (**b**) *s* = 0.4, (**c**) *s* = 0.6, (**d**) *s* = 0.8, (**e**) *s* = 1.0, (**f**) *s* = 1.2.

The stadium model was positioned at a ratio of 1:100 in a tornado center to investigate the beneficial effects of tornadoes on the stadium. The wind pressure coefficient diagrams of the stadium’s upper and lower surfaces with swirl ratios of 0.2, 0.4, and 0.6 are shown in Figs. 8 and 9. The stadium’s top and lower surfaces are all positive pressure when the swirl ratio is 0.2. The lower surface wind pressure system is 2.0, the minimum wind pressure system is 0.5, and there is a significant amount of wind pressure floating close to the fourth rib of the front eaves of the upper surface canopy. The airflow emerges as a vortex and flow separation in the third to fifth rib sections on the upper surface of the canopy when the swirl ratio is 0.4, indicating a significant negative pressure. The maximum negative wind pressure coefficient in the vortex area is − 1.4, and the maximum positive wind pressure coefficient on the upper surface reaches 0.6.

The stadium’s lower surface experiences positive pressure and the top surface’s fourth rib experiences negative pressure when the eddy current ratio is between 0.4 and 0.8. The stadium's fourth rib area is currently bearing a significant upward force and exhibiting "up suction down pressure." The stadium's lower surface experiences negative pressure when the swirl ratio is greater than or equal to 1.0, with a maximum value of − 0.6. The vortex's range expands and the negative pressure area at the canopy's fourth rib progressively rises when the swirl ratio is between 0.4 and 0.6. This causes a significant inverse pressure gradient and significant variations in the wind pressure coefficient. The maximum negative wind pressure coefficient is − 1.2 when the swirl ratio is 0.4, and the maximum negative wind pressure coefficient is − 2.2 when the swirl ratio is 0.6. The maximum negative pressure grows as the swirl ratio rises. The maximum negative pressure steadily drops from − 4.0 to  − 2.4 as the swirl ratio shifts from 0.8 to 1.2. The negative pressure diffuses from the fourth rib area to the upper surface of the entire canopy. The above results are mainly due to the increase of vortex core radius with the increase of vortex ratio. The updraft is skewed and rises vertically because the front eaves at the top of the canopy obstruct the airflow rising in the tornado's core. The tornado airflow is split into two pieces by the canopy obstacle, and the positive and negative pressure differences are produced at the negative pressure area of the canopy's front eaves.

### Comparison of wind pressure of stadium with different ground roughness

**Figure 10:**
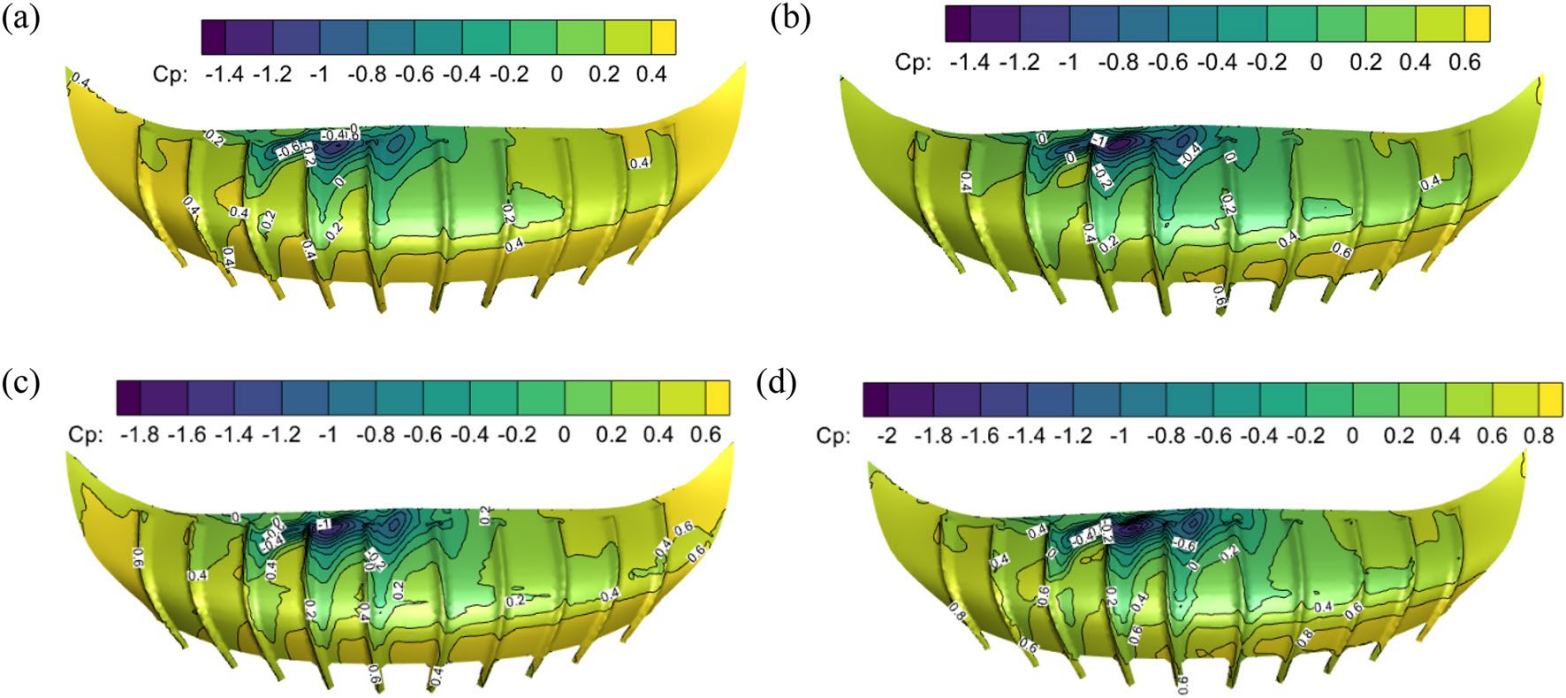
Wind pressure coefficient diagram of stadium in different ground roughness, (**a**) *K*_s_ = 0.6, (**b**) *K*_s_ = 2.0, (**c**) *K*_s_ = 10, (**d**) *K*_s_ = 40.

The wind pressure coefficient of the stadium under different ground roughness with swirl ratio of 0.4 is analyzed to explore the influence of ground roughness on the stadium. The wind pressure coefficient of the stadium in different landform roughness is shown in Fig. 10. The ground roughness is realized by equivalent ground roughness, and the ground roughness is set at the bottom of the tornado simulator. The logarithmic laws of the atmospheric boundary layer (ABL) and the wall function serve as the foundation^36^. The ground roughness height *K*_s_ and the roughness constant *C*_s_ value combine to produce the first-order continuous fitting. *K*_s_ = 20y_0_, *C*_s_ = 0.5. At *y*_0_ = 0.03 m for lower grass plains, *K*_s_ equals 0.6. For unpaved open ground, *y*_0_ = 0.1 m, followed by *K*_s_ = 2. When the ground is exceedingly rough,* y*_0_ = 0.5 m and *K*s = 10. For the urban center *y*_0_ = 2.0 m, therefore *K*_s_ = 40.

The stadium's wind pressure coefficient distribution varies when the swirl ratio remains constant but the landforms change. The stadium’s maximum positive pressure rises in comparison to the rough heights of 0.6 and 2, and the maximum wind pressure coefficient on open terrain surpasses 0.6 when compared to the grass plain. The maximum negative pressure of the canopy rises and the wind pressure coefficient rises from − 1.4 to − 1.8 on rugged terrain relative to open terrain, as indicated by the roughness heights of 2 and 10. The stadium’s location in the city center results in higher maximum positive and negative pressure as well as the highest wind pressure coefficient span when compared to roughness heights of 10 and 40. The radius of the vortex core diminishes and the extreme range of wind pressure increases as a result of the ground's roughness, which also lowers the wind field's maximum tangential velocity and reduces the swirl ratio^5,37^.

### Comparison of wind pressure and wind speed of stadium under different wind load conditions

**Figure 11:**
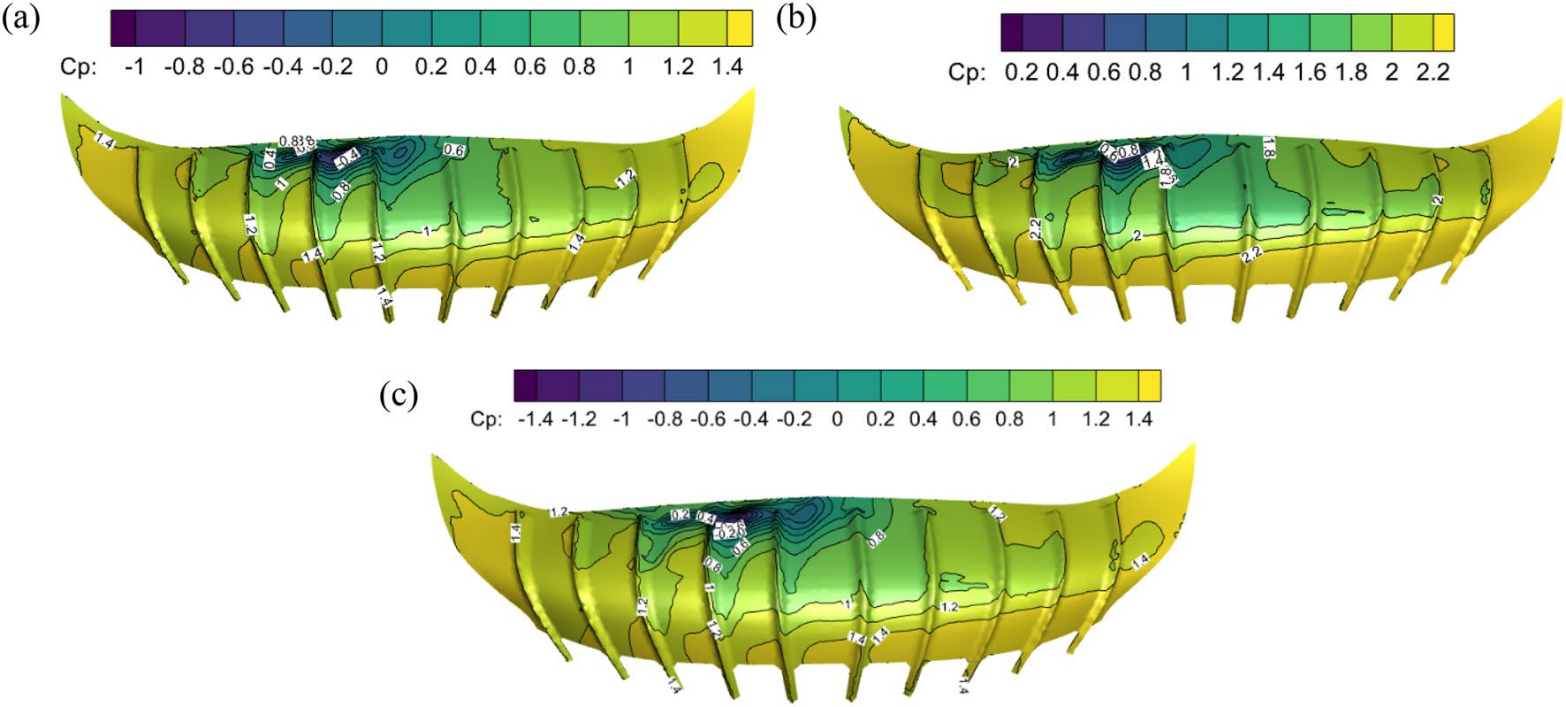
The wind pressure coefficient on the surface of the stadium with or without friction and wind profile. (**a**) No wind profile and no ground roughness, (**b**) wind profile and no ground roughness, (**c**) no wind profile and ground roughness.

Analysis of the effects of tornado wind profile and ground roughness on the wind pressure coefficient of the stadium is required since these factors have an impact on the radius and creation of the tornado vortex core. The swirl ratio of 0.4 and the rough open terrain *K*_s_ = 2 are selected. The comparison of the wind pressure coefficients on the upper surface of the stadium with or without wind profile and ground roughness is shown in Fig. 11. The wind pressure of the stadium with and without wind profile is compared and analyzed. The stadium's wind pressure is positive due to its wind profile. The minimum wind pressure coefficient is 0.2, and the maximum wind pressure coefficient is 2.2. The canopy without a wind profile has a minimum wind pressure coefficient of − 0.4 and a maximum wind pressure coefficient of 1.4. The maximum wind pressure coefficient of the wind profile is 0.8 larger than that of the windless profile, and the wind profile can improve the wind pressure coefficient of the stadium surface as a whole. The dense region of contour lines with ground roughness is larger when comparing and evaluating the stadium with and without ground roughness. The wind pressure coefficient has a wide range of values; its maximum negative value, − 1.4, is higher than the wind pressure coefficient without ground roughness. The main reason is that the ground roughness reduces the swirl ratio, thus the radius of the vortex core decreases^7^.

**Figure 12:**
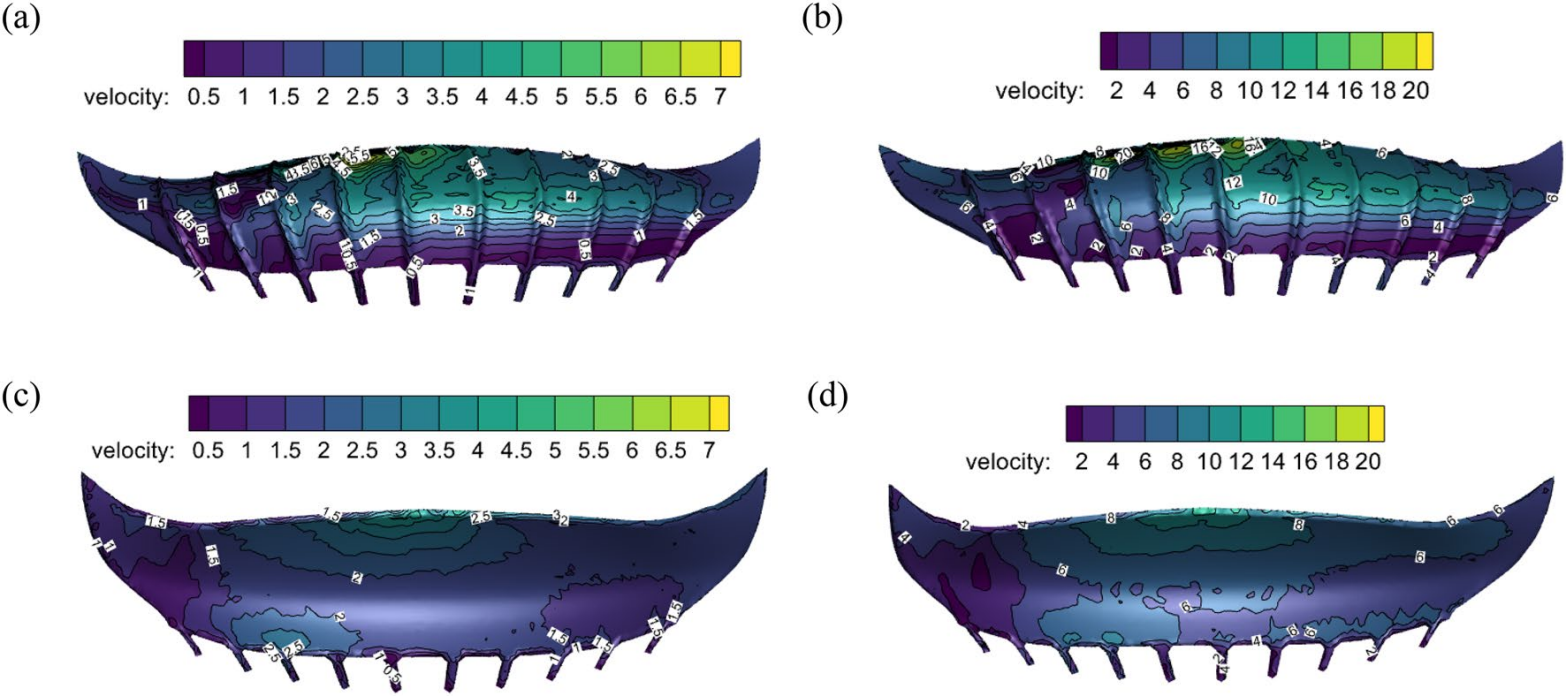
The velocity contours of the upper and lower surfaces of the stadium with different swirl ratio, (**a**) *s* = 0.2 upper surface wind speed, (**b**) *s* = 0.6 upper surface wind speed, (**c**) *s* = 0.2 lower surface wind speed, (**d**) *s* = 0.6 lower surface wind speed.

Figure 12 shows the velocity contours of the upper and lower surfaces of the stadium at different swirl ratio. The analysis shows that the local surface of the stadium is prone to large velocity. The maximum wind speed on the canopy's upper surface gradually rises as the swirl ratio does. The greatest wind speed near the fourth rib on the left side of the upper surface is 6.0 m/s when the swirl ratio is 0.2, and the minimum wind speed is 0.5 m/s. The upper surface wind speed is 20.0 m/s at its greatest and 2.0 m/s at its minimum when the swirl ratio is 0.6. The wind speed of the lower surface of the canopy is smaller than that of the upper surface, and the maximum wind speed appears in the front eaves of the canopy.

### Comparison of wind pressure on longitudinal section of stadium with different swirl ratios

**Figure 13:**
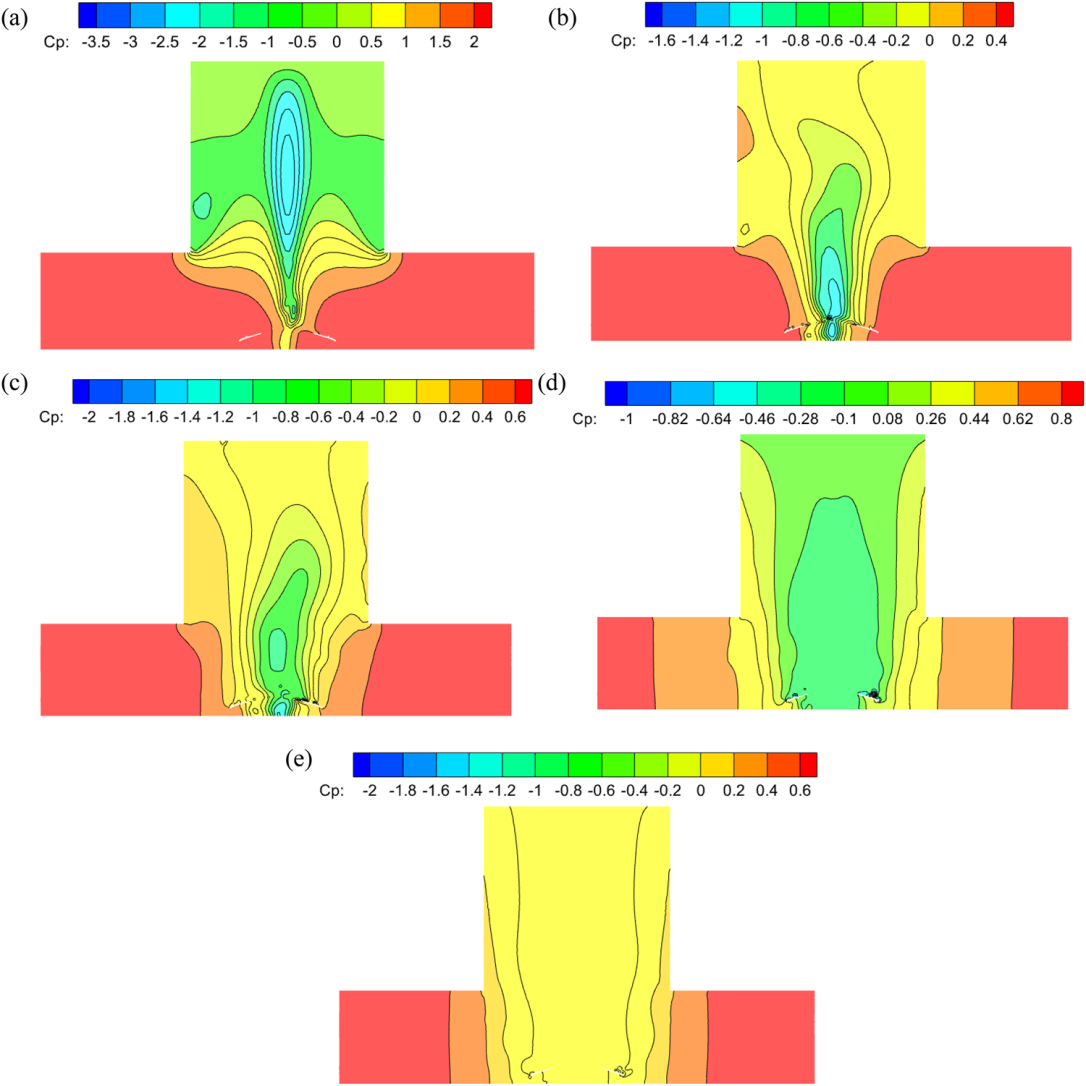
Longitudinal Sect. “Introduction” of stadium with different swirl ratio in wind field. (**a**) *s* = 0.2, (**b**) *s* = 0.6, (**c**) *s* = 1.0, (**d**) *s* = 0.8, (**e**) *s* = 1.0.

**Figure 14:**
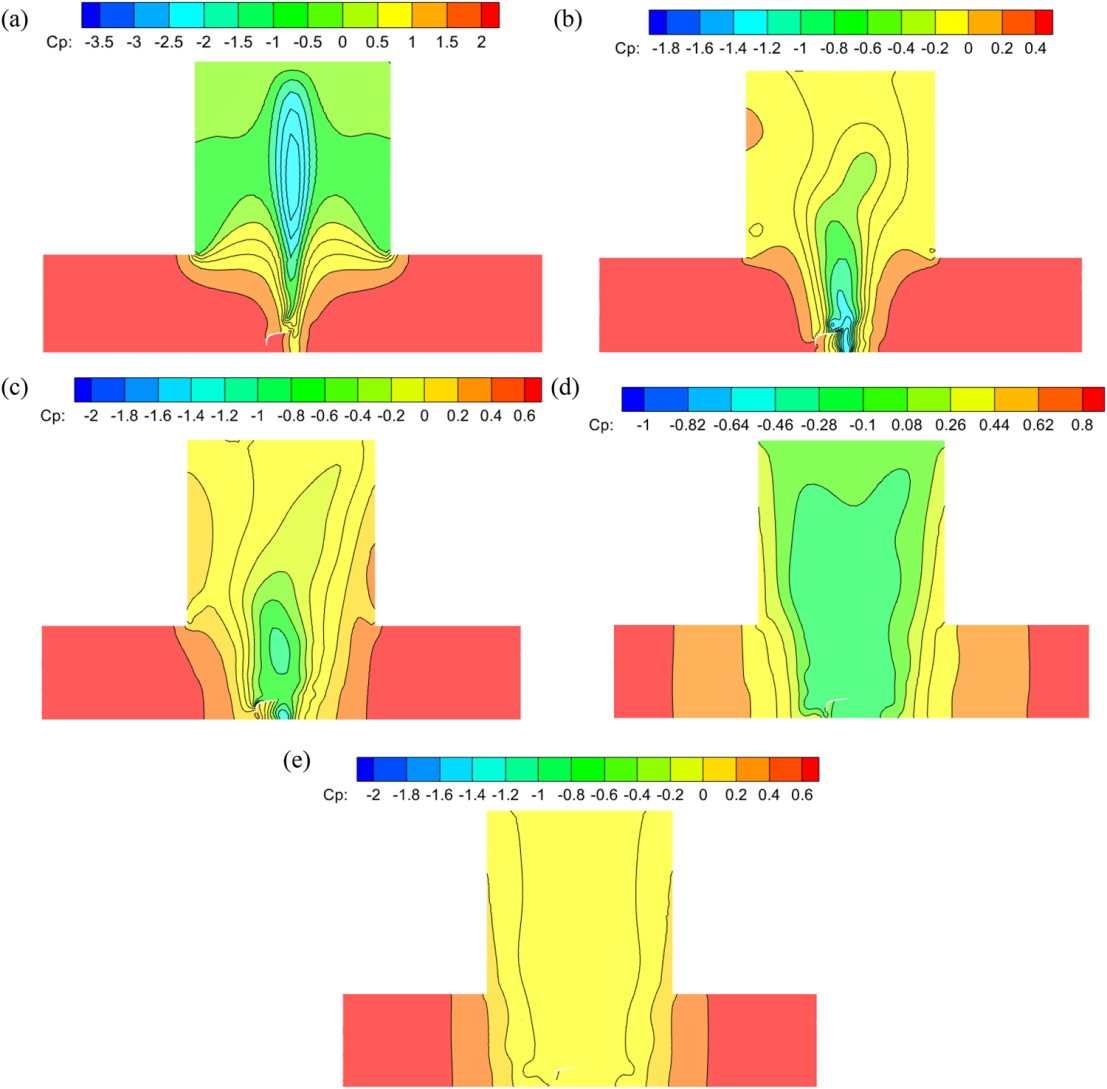
The longitudinal Sect. “Numerical simulation of tornado” of the stadium with different swirl ratio in the wind field. (**a**) *s* = 0.2, (**b**) *s* = 0.6, (**c**) *s* = 1.0, (**d**) *s* = 0.8, (**e**) *s* = 1.0.

The longitudinal section of the tornado generating device's centre was obtained for study in order to better investigate how the stadium affected the formation of the tornado vortex core under various swirl ratios. Figures 13 and 14 show the wind pressure coefficients of the vertical section of the stadium with different swirl ratios. Section “Introduction” is a cross-section along the stadium at the center of the vortex core, and Sect. “Numerical simulation of tornado” is a deep section along the stadium at the center of the vortex core. The lower half of the stadium and its surroundings experience positive pressure when the swirl ratio is 0.2 because the canopy is obstructing the tornado's updraft. The canopy's upper side experiences negative pressure, and the difference in pressure between positive and negative causes the front eaves to be unevenly stressed. This causes the vertical upward airflow to shift in these areas. The stadium splits the tornado vortex into upper and lower halves when the swirl ratio reaches 0.6. The stadium's two sides have the highest positive pressure, and the center of the canopy has the highest negative pressure. The lowest side of the stadium has the highest negative wind pressure coefficient when the swirl ratio is 1.0; this value is greater than the radius of the vortex core when the swirl ratio is between 0.2 and 0.6. The stadium is enveloped in a tornado vortex core with a strong reverse pressure gradient and vortex appearance on both sides of the canopy when the swirl ratio is less than or equal to 0.8. It is evident from the study above that the tornado's vortex core radius grows as the swirl ratio does.

### Different swirl ratio and roughness height stadium wind pressure coefficient and wind speed

**Figure 15:**
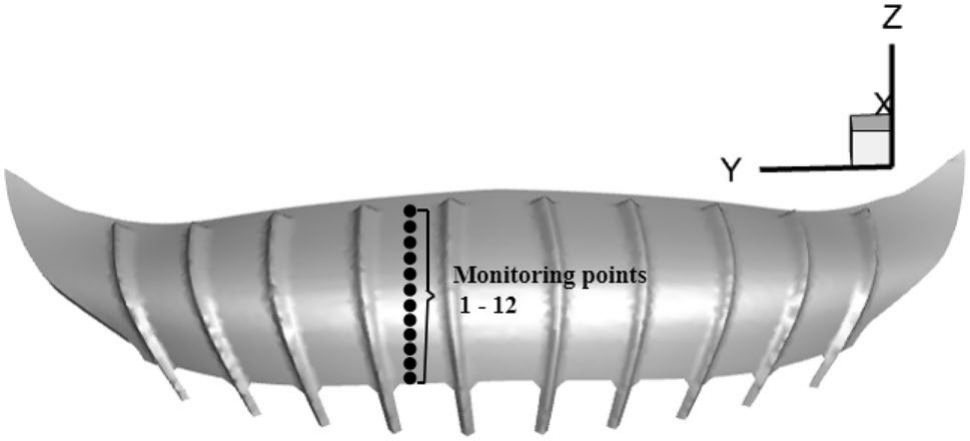
Schematic diagram of stadium monitoring points.

**Figure 16:**
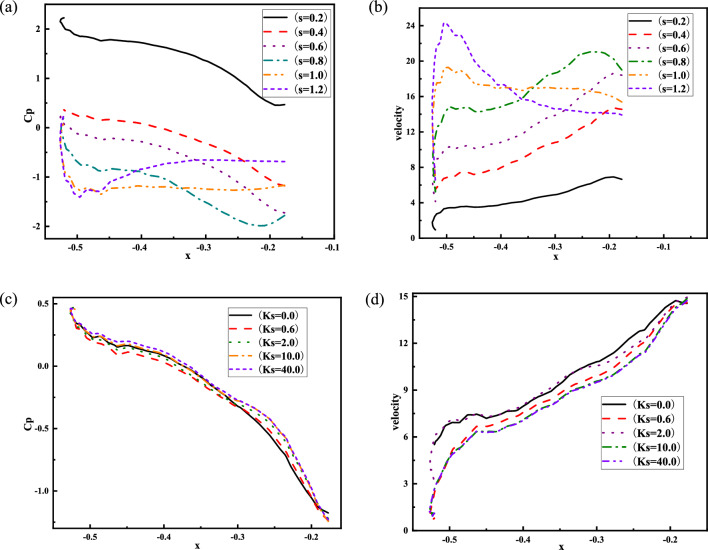
Different swirl ratio and roughness height stadium wind pressure coefficient and wind speed, (**a**) different swirl ratio wind pressure coefficient, (**b**) different swirl ratio wind speed, (**c**) different ground roughness wind pressure coefficient, (**d**) different ground roughness wind speed.

The surrounding spots are observed because of the significant variations in the wind pressure coefficient of the fourth rib on the left side of the stadium. Figure 15 shows 12 monitoring points on the surface of the stadium. The wind pressure coefficients and wind speeds of different swirl ratios and roughness heights are shown in Fig. 16. It is evident from comparing the wind pressure and velocity of various swirl ratios that the monitoring sites are positive pressure when the swirl ratio is 0.2. The absolute value of the wind pressure coefficient and the wind speed increase gradually with an increase in x when x ≥ − 0.45 (x is the abscissa of the monitoring point, which measures the distance from the core) and the swirl ratio is between 0.4 and 1.2. The absolute value of the wind pressure coefficient and the wind speed steadily drop with an increase in x when x > − 0.45 and the swirl ratio is greater than 0.8. The wind pressure coefficient and wind speed progressively rise as the swirl ratio increases when it is less than 0.8. The wind pressure coefficient and wind speed on the canopy's surface far from the core gradually decrease as the swirl ratio rises to greater than 0.8, while the wind pressure coefficient and wind speed on the canopy's surface close to the core gradually increase. It is evident from the comparison of wind pressure and varying ground roughness that the absolute value of the wind pressure coefficient steadily declines with increasing ground roughness when x > − 0.45. The wind pressure coefficient is comparable to the wind speed between roughness heights of 10 and 40. Comparing the wind speed of different ground roughness, it is found that the wind speed gradually decreases with the increase of ground roughness height. The radius of the vortex core, wind pressure coefficient, wind speed, and wind field's influence on the canopy are all lowered as a result of ground roughness's effect on lowering the swirl ratio.

### Vortex diagram of different swirl ratio

**Figure 17:**
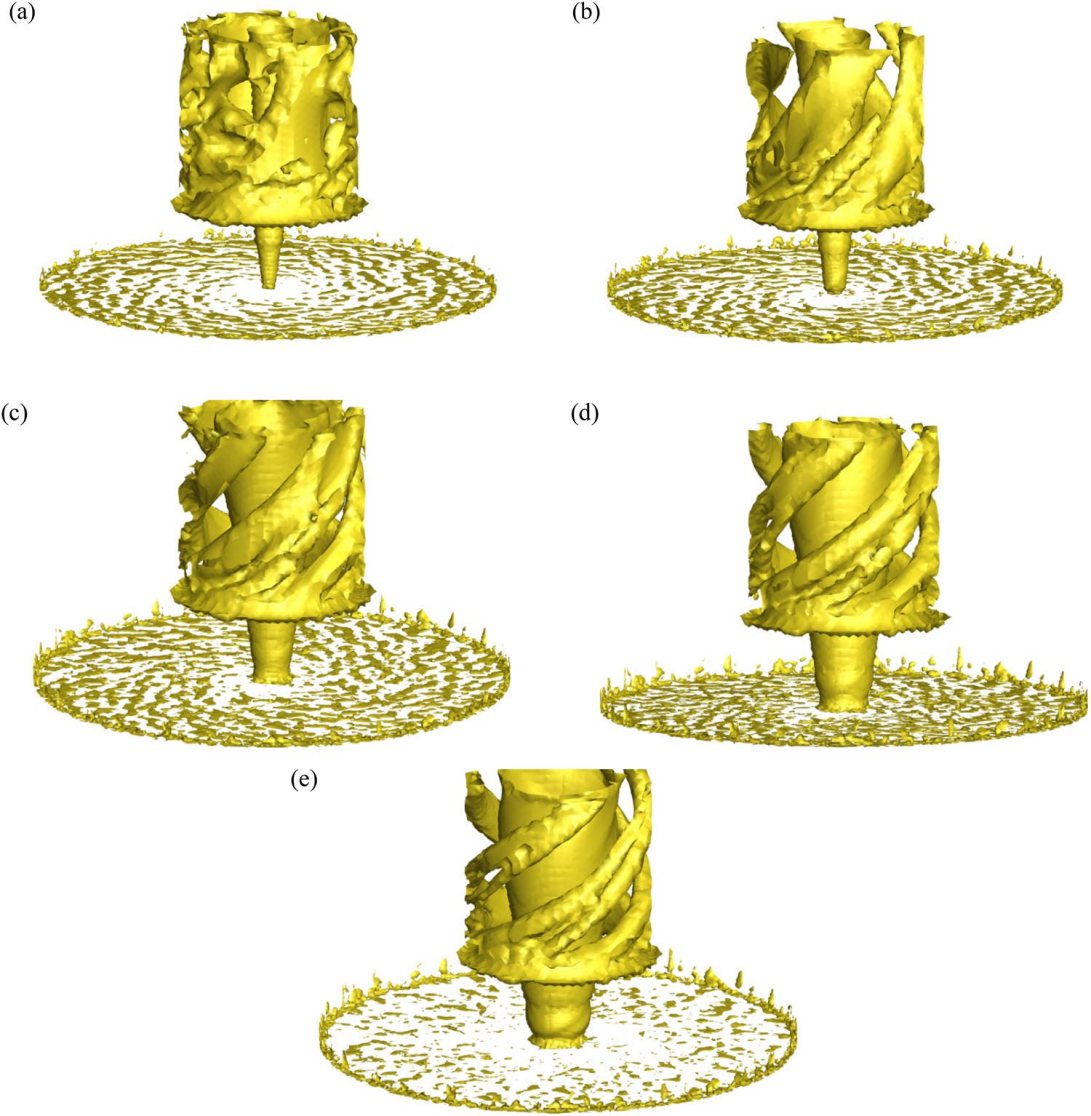
Vortex volume of tornadoes without stadium with different swirl ratios, (**a**) *s* = 0.2, (**b**) *s* = 0.4, (**c**) *s* = 0.6, (**d**) *s* = 0.8, (**e**) *s* = 1.0.

**Figure 18:**
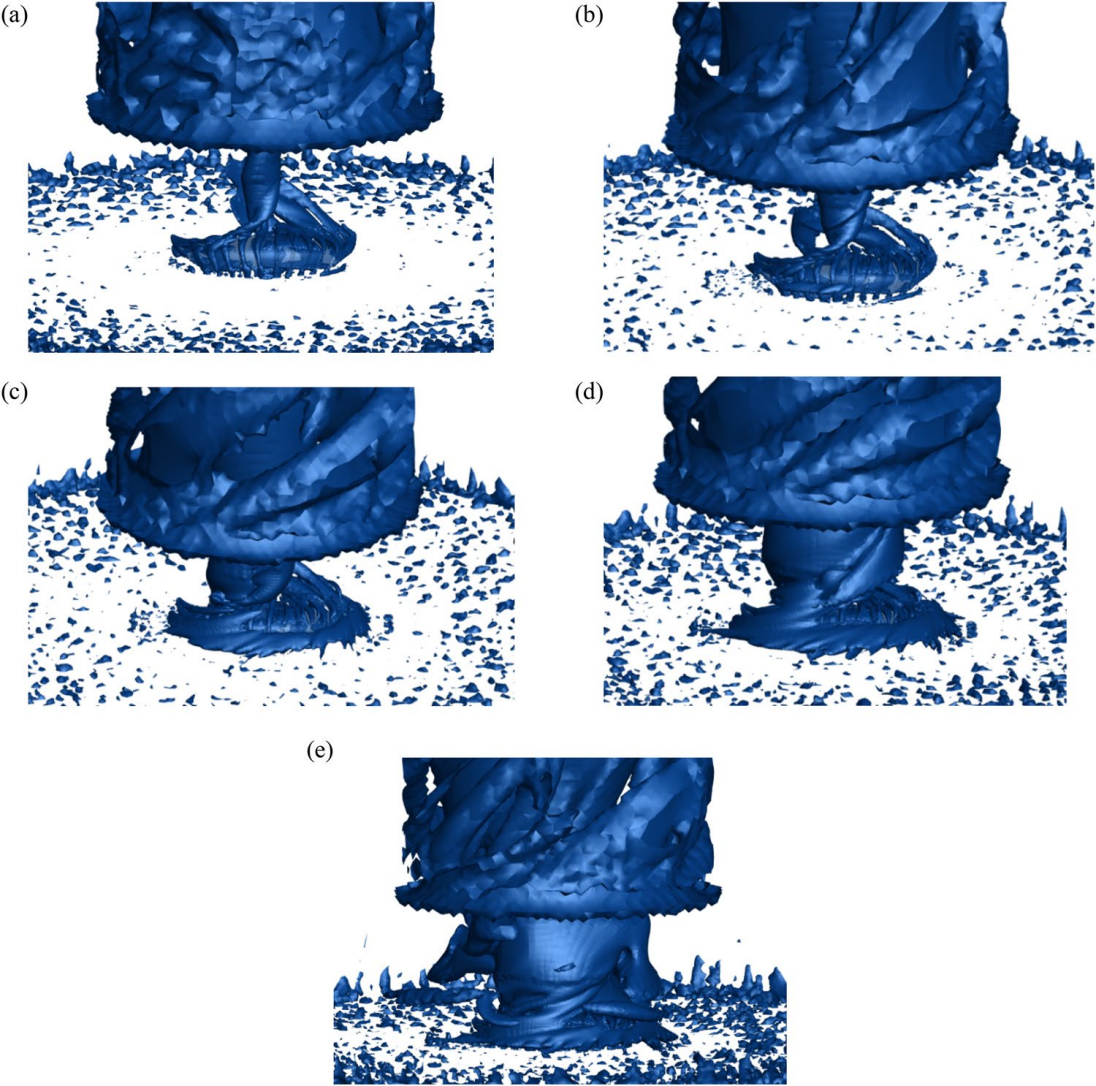
Vortex distribution map around the stadium with different swirl ratios, (**a**) *s* = 0.2, (**b**) *s* = 0.4, (**c**) *s* = 0.6, (**d**) *s* = 0.8, (**e**) *s* = 1.0.

This research studies the vorticity distribution using the Q criterion (threshold of 0.01), which is useful for examining the formation process of tornado vortices. The wind field without stadium and the vorticity around the stadium at different swirl ratio are shown in Figs. 17 and 18, respectively. The diameter of the tornado core vortex progressively increases in the non-stadium wind field as the swirl ratio rises. Vorticity rises when the swirl ratio rises in the wind field containing the stadium. The stadium will be fully engulfed in the vortex when the swirl ratio reaches 1.0. The stadium's vortex diameter is greater than the non-stadium's when the swirl ratio is the same. The vortex is affixed to the canopy's surface and rises along the stadium's construction. The original vertical upward airflow altered and then climbed again as a result of the awning's destruction of the tornado's original vortex core growth and obstruction of upward vorticity. The position where vorticity first appears is in the rib and bottom support area of the stadium. The stadium's upper surface experiences vortex shedding when the swirl ratio reaches 0.4. The quantity of vortex shedding close to the ground increases as the swirl ratio rises, the vortex exhibits an upward spiral pattern, and the rising vortex's diameter progressively grows. The vortex is guided by the canopy's front eaves, causing the vorticity to extend forward along the canopy's ribs.

## Conclusion

This research presents a numerical simulation of the stadium's wind load characteristics during a tornado. The wind pressure coefficient distribution, velocity distribution, ground roughness, and vorticity on the stadium's upper and lower surfaces were examined based on varying swirl ratios. The following conclusions are obtained:

1. The stadium's top rib section on the upper surface displays negative pressure and the stadium's lower surface is positive pressure when the swirl ratio shifts from 0.4 to 0.8. The negative pressure area presents’ up suction and down pressure’, and the canopy bears a large upward force and is vulnerable to damage. The wind pressure coefficient and wind speed increase steadily as the swirl ratio increases when it is less than or equal to 0.8. The wind pressure coefficient and wind speed gradually drop as the swirl ratio increases when it is more than 0.8.
2. The radius of the vortex core, the wind pressure coefficient, the wind speed, and the damage caused by the wind field to the canopy all diminish when the ground roughness lowers the swirl ratio. The wind pressure and wind speed progressively decrease as the roughness height rises. The windy section's maximum wind pressure coefficient is 0.8 higher than the windless section’s when the swirl ratio is 0.4. The positive and negative pressure differential on the stadium's surface increases due to the wind profile, making the canopy more prone to damage.
3. The airflow is disrupted by the ribs at the top of the stadium canopy, causing a small vortex to form on the leeward side of the ribs that displays a windward pressure gradient. The wind field's core vortex is split into two sections by the stadium, with the bottom section having a lesser wind speed. It is mainly horizontal rotation, and the upper part is mainly vertical upward, and the upward speed is larger.
4. The diameter of the tornado core vortex steadily expands in the wind field without stadium as the swirl ratio increases. The wind field with the stadium has a bigger core vortex diameter than the wind field without the stadium at the same swirl ratio. The airflow is skewed and then rises vertically as a result of the front eaves at the top of the canopy blocking the updraft. The aerodynamic force and the stadium at different positions in the wind field are not explored in this paper. The interaction between the tornado movement and the structure and wind field is also not considered. These issues will be the focus of our next study.

## Data Availability

The datasets used or analysed during the current study available from the corresponding author Z.Z on reasonable request.
